# Antiplasmodial Activity, Cytotoxicity and Structure-Activity Relationship Study of Cyclopeptide Alkaloids

**DOI:** 10.3390/molecules22020224

**Published:** 2017-02-02

**Authors:** Emmy Tuenter, Karen Segers, Kyo Bin Kang, Johan Viaene, Sang Hyun Sung, Paul Cos, Louis Maes, Yvan Vander Heyden, Luc Pieters

**Affiliations:** 1 Natural Products & Food Research and Analysis (NatuRA), Department of Pharmaceutical Sciences, University of Antwerp, Universiteitsplein 1, 2610 Antwerp, Belgium; Luc.Pieters@uantwerpen.be; 2 Department of Analytical Chemistry and Pharmaceutical Technology, Center for Pharmaceutical Research (CePhaR), Vrije Universiteit Brussel—VUB, Laarbeeklaan 103, B-1090 Brussels, Belgium; Karen.Segers@vub.ac.be (K.S.); Johan.Viaene@vub.ac.be (J.V.); yvanvdh@vub.ac.be (Y.V.H.); 3 College of Pharmacy and Research Institute of Pharmaceutical Sciences, Seoul National University, 1 Gwanak-ro, Gwanak-gu, Seoul 08826, Korea; mumyung2@snu.ac.kr (K.B.K.); shsung@snu.ac.kr (S.H.S.); 4 Laboratory of Microbiology, Parasitology and Hygiene (LMPH), Faculty of Pharmaceutical, Biomedical and Veterinary Sciences, University of Antwerp, Universiteitsplein 1, 2610 Antwerp, Belgium; Paul.Cos@uantwerpen.be (P.C.); Louis.Maes@uantwerpen.be (L.M.)

**Keywords:** cyclopeptide alkaloids, antiplasmodial activity, cytotoxicity, SAR

## Abstract

Cyclopeptide alkaloids are polyamidic, macrocyclic compounds, containing a 13-, 14-, or 15-membered ring. The ring system consists of a hydroxystyrylamine moiety, an amino acid, and a β-hydroxy amino acid; attached to the ring is a side chain, comprised of one or two more amino acid moieties. In vitro antiplasmodial activity was shown before for several compounds belonging to this class, and in this paper the antiplasmodial and cytotoxic activities of ten more cyclopeptide alkaloids are reported. Combining these results and the IC_50_ values that were reported by our group previously, a library consisting of 19 cyclopeptide alkaloids was created. A qualitative SAR (structure-activity relationship) study indicated that a 13-membered macrocyclic ring is preferable over a 14-membered one. Furthermore, the presence of a β-hydroxy proline moiety could correlate with higher antiplasmodial activity, and methoxylation (or, to a lesser extent, hydroxylation) of the styrylamine moiety could be important for displaying antiplasmodial activity. In addition, QSAR (quantitative structure-activity relationship) models were developed, using PLS (partial least squares regression) and MLR (multiple linear regression). On the one hand, these models allow for the indication of the most important descriptors (molecular properties) responsible for the antiplasmodial activity. Additionally, predictions made for interesting structures did not contradict the expectations raised in the qualitative SAR study.

## 1. Introduction

With an estimated amount of 214 million new cases in 2015, malaria is still one of the most important infectious parasitic diseases worldwide [1]. It is caused by the *Plasmodium* parasite, and it is transferred via the bite of an *Anopheles* mosquito. Because of increasing resistance against the currently available antimalarial drugs, there is a compelling need for new therapeutic agents. It is well-recognized that plants are interesting sources for identifying new lead compounds [2], which is well-exemplified by the alkaloid quinine or the terpene artemisinine.

This manuscript focuses on a particular subclass of alkaloids, namely the cyclopeptide alkaloids. These are macrocyclic compounds, containing a 13-, 14-, or 15-membered ring. The ring consists of a hydroxystyrylamine moiety, a typical amino acid, and a β-hydroxy amino acid; attached to the ring is a side chain, usually comprised of one or two more amino acid moieties. Their basic character is related to the N-atom of the terminal amino acid moiety in the side chain; however, in some cases a cinnamoyl instead of an amino acid moiety is present, and this type of cyclopeptide alkaloid is referred to as a “neutral cyclopeptide alkaloid”.

Certain plants containing cyclopeptide alkaloids are used in traditional medicine for the treatment of malaria, for example, the leaves and roots of *Hymenocardia acida* [3]. The in vitro antiplasmodial activity of 19 cyclopeptide alkaloids has been published so far. Suksamrarn et al. found antiplasmodial activity for ziziphines-N and -Q (IC_50_ values of 3.92 and 3.5 μg/mL, corresponding to 6.4 and 5.9 μM, respectively), while ziziphines-O and -P were not active [4]. Panseeta et al. identified mauritine-M, nummularine-H, and hemsine-A as antiplasmodial compounds, with IC_50_ values ranging between 3.7 and 7.3 μM. Nummularine-B was found to be moderately active (IC_50_ value of 10.3 μM) against *Plasmodium falciparum*, but not mauritine-L and nummularine-B methiodide [5]. Our research group has previously reported the antiplasmodial activity of cyclopeptide alkaloids from *Hymenocardia acida* and *Ziziphus oxyphylla* [6,7]. Based on these results, some preliminary conclusions concerning their SAR (structure-activity relationship) were drawn, but given the fact that the number of compounds tested in each of these studies was relatively small, it was not possible to draw definite conclusions. In the present paper, the antiplasmodial and cytotoxic activities of ten cyclopeptide alkaloids are reported, of which only the antiplasmodial activity of nummularine-B was reported previously [5]. These results were then combined with previous data obtained by our group in the same test conditions for nine other cyclopeptide alkaloids from *H. acida* and *Z. oxyphylla* [6,7], in order to perform a SAR study, and to identify the molecular characteristics that can account for high antiplasmodial activity.

## 2. Results and Discussion

### 2.1. Antiplasmodial and Cytotoxic Activities

The in vitro antiplasmodial activity against *P. falciparum* strain K1 and the cytotoxicity against MRC-5 cells was determined for ten cyclopeptide alkaloids, isolated from the stem bark of *Ziziphus nummularia*, *Ziziphus spina-christi*, *Ziziphus jujuba*, and the root of *Hovenia dulcis*, all belonging to the Rhamnaceae family, and the results are shown in Table 1. The results that were previously reported for cyclopeptide alkaloids isolated from the root bark of *H. acida* (Phyllanthaceae) and the roots of *Z. oxyphylla* (Rhamnaceae) [6,7] are also included in this table. The highest antiplasmodial activity was found for spinanine-B (**3**), with an IC_50_ value of 2.1 μM and without cytotoxic effects in concentrations of 64.0 μM or less. Nummularine-B (**8**) also showed promising antiplasmodial activity (IC_50_ value of 3.6 μM), and did not have any cytotoxic effects up to a concentration of 64.0 μM. However, Panseeta et al. [5] previously considered this compound to be only moderately active (IC_50_ value of 10.3 μM).

### 2.2. Qualitative Structure-Activity Relationship Study

**Figure 1:**
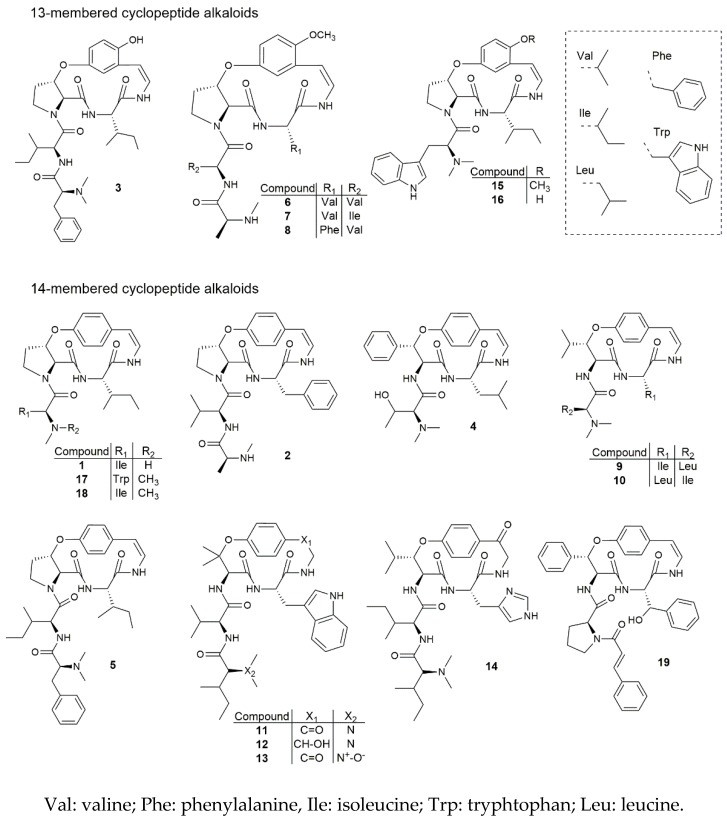
Chemical structures of cyclopeptide alkaloids **1–19**.

The chemical structures of all 19 cyclopeptide alkaloids are shown in Figure 1. Based on these structures and the results obtained for the antiplasmodial activity, a preliminary structure-activity relationship study was performed. From the 19 cyclopeptide alkaloids tested, eight had an IC_50_ value below 10 μM. Out of these eight, five belonged to the 13-membered and three to the 14-membered cyclopeptide alkaloids. Interestingly, the five most promising results were found for cyclopeptides bearing a 13-membered ring, with IC_50_ values ranging between 2.1 and 7.1 μM. From these results, it could be concluded that a meta-cyclophane structure (13-membered ring) is more likely to be associated with a high antiplasmodial activity compared to a para-cyclophane structure (14-membered ring). Moreover, out of the six tested 13-membered cyclopeptide alkaloids, only one showed an IC_50_ value above 10 μM, namely jubanine-F (**6**) (IC_50_ value of 12.8 μM), but this result still indicates moderate activity. Thus, all tested meta-cyclophane compounds were found to be antiplasmodially active. As for the 14-membered cyclopeptide alkaloids, only three of the 13 tested compounds were found to have an IC_50_ value below 10 μM, while nummularine-E (**4**) could not inhibit *P. falciparum* in concentrations up to 64.0 μM. These findings support our hypothesis that a 13-membered ring is favorable regarding the high antiplasmodial activity.

When looking further into the compounds containing a 13-membered macrocyclic ring, two contain a styrylamine moiety, hydroxylated in position 2 of the aromatic ring, while the other four possess a 2-methoxy styrylamine moiety. With respect to this structural feature, Suksamrarn et al. [4], who assessed the antiplasmodial activity of four 13-membered cyclopeptide alkaloids, proposed that the methoxy group would be crucial for displaying antiplasmodial activity. Our results do not support this, since the tested hydroxylated meta-cyclophane compounds were also found to be active, but interestingly, compound **15** (nummularine-R, IC_50_ value of 3.2 μM) seemed to be more active than compound **16** (*O*-desmethylnummularine-R, IC_50_ value of 7.1 μM), with their only difference being the presence of a hydroxy or a methoxy group, respectively. Based on this result, it might be concluded that cyclopeptide alkaloids containing either a methoxy group or a hydroxy group can display antiplasmodial activity, but that methoxylated compounds might be associated with higher activity. In view of this, it would be interesting to determine, for example, the antiplasmodial activity of paliurine-C, which is the methoxylated analogue of spinanine-B. Moreover, it would be interesting to test the 13-membered cyclopeptide alkaloids without a methoxy or hydroxy group, in order to know how this affects their antiplasmodial activity. However, to the best of our knowledge, such cyclopeptide alkaloids have not been reported up to now [8], although they could possibly be obtained by semi-synthesis.

One common feature of all tested meta-cyclophane compounds is the presence of a β-hydroxy proline amino acid moiety. Panseeta et al. [5] suggested that this feature could be linked to a relatively high antiplasmodial activity. They found promising results for the 13-membered cyclopeptide alkaloids mauritine-M and nummularine-H, and for hemsine-A (**17**), the latter belonging to the para-cyclophane compounds. In our case, all para-cyclophane compounds bearing a β-hydroxylated proline showed only moderate or weak activity against *P. falciparum*, except for amphibine-D (**5**) (IC_50_ value of 8.9 μM). Moreover, the 14-membered cyclopeptide alkaloids comprised of a β-hydroxy phenylalanine (oxyphylline-F) (**19**) or a β-hydroxy leucine (adouetine-X) (**9**) showed promising antiplasmodial activities. These results indicate that a β-hydroxy proline moiety is not crucial for displaying antiplasmodial activity. In order to confirm whether this finding is applicable to the 13-membered cyclopeptide alkaloids as well, it would be valuable to know to what extent meta-cyclophane cyclopeptide alkaloids with different β-hydroxy amino acid moieties are capable of inhibiting the growth of the *Plasmodium* parasite. However, to the best of our knowledge, all reported 13-membered cyclopeptide alkaloids contain such a β-hydroxy proline moiety; thus, for the moment, it is not possible to determine the activity of other types of meta-cyclophane cyclopeptide alkaloids.

Another interesting finding is the difference in activity between adouetine-X (**9**) (IC_50_ value of 7.5 μM) and frangulanine (**10**) (IC_50_ value of 14.9 μM). These two compounds show many structural similarities, and differ only in two aspects: adouetine-X contains an isoleucine moiety as a ring-bound amino acid, while frangulanine contains a leucine moiety in this position. The opposite is applicable for the side chain, with a leucine unit found in adouetine-X and an isoleucine moiety found in frangulanine. Leucine and isoleucine are constitutional isomers, hence, a small difference in chemical structure can affect the antiplasmodial activity. Suksamrarn et al. [4] concluded that substitution of a leucine unit with a valine unit, both aliphatic amino acid units as well, resulted in similar biological activities. In their case, the substitution took place in the side chain (ziziphine-N vs. ziziphine-Q). The cyclopeptide alkaloids jubanines-F (**6**) and -G (**7**) also differ only in one amino acid unit in the side chain, with jubanine-F containing a valine moiety and jubanine-G an isoleucine moiety. However, the antiplasmodial activity found for jubanine-G (IC_50_ value of 4.7 μM) was significantly higher than the activity for jubanine-F (IC_50_ value of 12.8 μM). Thus, the statement made by Suksamrarn et al. [4] is not supported by our results, and it must be concluded that the substitution of one aliphatic amino acid with another does affect the resulting antiplasmodial activity.

### 2.3. Quantitative Structure-Activity Relationship Study

In order to have a quantitative understanding of the structure-activity relationships underlying some of the compounds’ selectivity with respect to antiplasmodial activity, a QSAR (quantitative structure-activity relationship) study was carried out. PLS (partial least squares regression) and MLR (multiple linear regression) models were built, linking a set of molecular descriptors with the antiplasmodial activity. Different data pretreatments, i.e., autoscaling, direct orthogonal signal correction (DOSC) [9], and their combinations were first applied to the descriptor data. DOSC was found to result in the best PLS model. The MLR models were built by selecting the descriptors based on their importance in the best DOSC-PLS model. Three models, with three, six, or eight descriptors were built, and revealed which molecular descriptors were of main importance for the antiplasmodial activity. The model with eight descriptors performed similarly well as the best PLS model.

The RMSECV (root mean squared error of cross validation) and RMSEC (root mean squared error of calibration) values of each model were calculated, in order to select the best model. For the best DOSC-PLS model, the RMSECV was 0.0134 (and the RMSEC was 0.0051). The more descriptors included in the MLR models, the lower the RMSECV value found. For the model built with three descriptors, the RMSECV was 0.4306 (RMSEC was 0.2150); for that with six descriptors it was 0.0725 (0.0505); and when eight descriptors were included it was 0.0257 (0.0095), respectively. However, the four models predicted the activity of the calibration samples well, even when they were considered as unknown compounds in the cross validation. For instance, the following MLR equation (eight-descriptor model) can be applied in order to have an estimation of the IC_50_ value obtained in the antiplasmodial activity assay:

$$\begin{array}{cc} IC_{50}\;\mathrm{value}(\mu M) & =0.0002+0.0436*\text{Platt index}+0.0345*\mathrm{MMFF}94\;\mathrm{energy}\\ & \;+0.0203*\text{Dreiding energy}+0.0188*\text{Maximal projection area}\\ & \;+0.0104*\text{Minimal projection area}+0.0105*\text{Wiener polarity}\\ & \;+0.0088*C(\%)+0.0089*\text{Aliphatic bond count} \end{array}$$

The eight molecular descriptors that were identified as most influential (in sequence added in the three MLR models) were: the Platt index, a path-based topological descriptor which is defined as the sum of the edge degrees of a molecular graph, and two geometrical descriptors, MMFF94 (Merck molecular force field 94) energy and the Dreiding energy (both given in kcal/mol), which are measures for the internal energy of a given conformer. In addition, two other geometrical descriptors, i.e., the maximal and minimal projection area (given in Å^2^); furthermore, the Wiener polarity, which represents the number of three bond length distances in the molecule (topological descriptor); the percentage of C-atoms in the molecule, and finally the aliphatic bond count. For all descriptors in the model, a higher value is associated with a higher IC_50_ value, and thus lower antiplasmodial activity. Unfortunately, the theoretical descriptors cannot always easily be linked to the physicochemical properties of the molecule.

Additionally, the models were used to predict the activity of a number of potentially interesting structures, as indicated by the qualitative SAR study. We stated earlier that it would be interesting to determine the antiplasmodial activity of paliurine-C, the methoxylated analogue of spinanine-B, as well as of the 13-membered cyclopeptide alkaloids without a methoxy or hydroxy group. The models built predict that both paliurine-C and 13-membered cyclopeptide alkaloids without a methoxy group would have rather high activities. Moreover, the importance of the β-hydroxy proline moiety in 13-membered cyclopeptide alkaloids was questioned, and it would be valuable to know to what extent meta-cyclophane cyclopeptide alkaloids with different β-hydroxy amino acid moieties are capable of inhibiting *P. falciparum*. Thus, a prediction of the activity of 13-membered cyclopeptide alkaloids bearing either a β-hydroxy leucine, β*-*hydroxy valine, or a β-hydroxy phenylalanine moiety was made, although such compounds have not been identified from natural sources yet. The theoretical descriptors derived from their structures allowed the prediction of a high activity for these compounds. In addition, the models seem to confirm the high activity for the 13-membered-ring compounds. When interpreting the predictions, it has to be taken into account that the calibration set was small (19 compounds), and that the structures for which the activities were predicted may be sub-optimally represented in it. Moreover, the data set mainly consisted of active and intermediately active compounds, while inactive compounds are underrepresented. Therefore, in our opinion, the interpretation of the activity at this moment, should or could not go further than the indication of whether or not a given compound is expected to be rather active or inactive. Better predictive models for these types of compounds will require a larger, more representative calibration set.

The chemical structures of promising cyclopeptide alkaloids defined in the qualitative discussion of this paper allowed the prediction of high activities using any of the QSAR models. The QSAR approach thus did not contradict the expectations gained from the qualitative study. In the future, promising compounds could be isolated from natural sources or constructed (semi-) synthetically, after which testing of their antiplasmodial activity can be performed.

## 3. Materials and Methods

### 3.1. General Experimental Procedures

Marvinsketch version 16.10.31.0 (Chemaxon, Budapest, Hungary) was used for obtaining the set of 73 molecular descriptors, and the QSAR models were developed in Matlab™ version 7.1 (The Mathworks, Natick, MA, USA).

### 3.2. Plant Material, Extraction, and Isolation

The extraction, isolation, and structure elucidation of 17 of the 19 cyclopeptide alkaloids included in this manuscript were reported previously [6,7,10,11]. The isolation procedure of adouetine-X and fragulanine is briefly described here:

Adouetine-X (25.1 mg) was isolated from the roots of *Ziziphus jujuba*. The roots were collected in JinJu, Korea, in 2012. The plant was identified by Prof. Eun Ju Jeong (Gyeongnam National University of Science and Technology, JinJu, Korea). A voucher specimen (SUPH-1204-01) was deposited in the Herbarium in the Medicinal Plant Garden, College of Pharmacy at Seoul National University in Korea. The dried and powdered plant material (14.5 kg) was macerated with methanol and the obtained crude extract was fractionated by liquid-liquid partitioning. Further fractionation of the alkaloid fraction was accomplished by means of column chromatography on a silica column (Kieselgel 60 silica gel (40–60 μm, 230–400 mesh, Merck, Darmstadt, Germany)) and a Sephadex column (Sephadex LH-20 (25–100 μm, Pharmacia, Piscataway, NJ, USA)), consecutively, as described by Kang et al. (2015). By means of preparative HPLC, adouetine-X was isolated from fraction A2c. The preparative HPLC system consisted of a G-321 pump (Gilson, Middleton, WI, USA), and a G-151 UV detector (Gilson). An XBridge C_18_ column (250 mm x 10 mm i.d.; 5 μm, Waters, Milford, MA, USA) was used to obtain separation (0.1% NH_4_Ac in H_2_O-acetonitrile, 5:5, 4 mL/min). Structure elucidation was performed on the basis of NMR (nuclear magnetic resonance) and HRESIMS (high resolution electrospray ionization mass spectrometry) experiments and comparison of the spectroscopic data to the literature [12].

Frangulanine was isolated from the methanolic extract of *Hovenia dulcis* roots. The plant material was cultivated at the Medicinal Plant Garden, College of Pharmacy, Seoul National University, Koyang, Korea, and was collected in September 2015. A voucher specimen (SUPH-1509-03) was deposited in the Herbarium of the Medicinal Plant Garden. Powdered, dried roots of *H. dulcis* (2.7 kg) were extracted with methanol by ultrasonication at room temperature (2 × 12 L, for 3 h each). The crude extract (87.1 g) was suspended in water and was partitioned successively with dichloromethane (17.7 g), ethyl acetate (22.0 g), and butanol (33.5 g). The CH_2_Cl_2_ fraction was subjected to silica gel column chromatography and eluted with mixtures of CHCl_3_−CH_3_OH (100:1, 50:1, 20:1, 15:1, 10:1, 5:1, and 3:1) to yield 10 fractions (MC1−MC10). Subfraction MC5 was further divided into six subfractions (MC5a−MC5f) by passage over Sephadex LH-20, eluted with a mixture of CH_2_Cl_2_−CH_3_OH (3:1). The CH_3_OH-insoluble precipitate of subfraction MC5d was collected and purified by washing with CH_3_OH, and was identified as frangulanine (28.3 mg) by comparison of its spectroscopic data (NMR, HRESIMS) to the data reported in the literature [13].

### 3.3. Antiplasmodial and Cytotoxic Activities

The antiplasmodial and cytotoxic activity determinations of all cyclopeptide alkaloids were performed as reported before [14,15]. Each experiment was performed at least in triplicate and the average IC_50_ values were calculated.

### 3.4. QSAR

Molecular models of all cyclopeptide alkaloids were generated using Marvinsketch. For the generation of the lowest energy conformer, the conformer plugin was used and the following settings were applied: Force field: Dreiding; optimization limit: very strict; prehydrogenize. Then, for each compound, 73 molecular descriptors were calculated, related to the elemental analysis, protonation, partitioning, solubility, topological analysis, geometrical properties, polar and molecular surface area, H-bond donor/acceptor characteristics, and refractivity. Two more descriptors were added, i.e., the number of subunits and N-methyl groups, since they were considered possibly important in relation to the bioactivity of the cyclopeptide alkaloids.

Prior to the actual molecular modelling, the descriptors which showed >95% correlation or which were constant were identified. The latter were deleted and of the former, the one that was best correlated to the activity was kept. This resulted in 41 remaining descriptors. Based on the set of molecular descriptors and the average IC_50_ values obtained in the antiplasmodial activity assay, partial least squares (PLS) and multiple linear regression (MLR) modelling were carried out. Models were selected on the basis of leave one out cross validation. The RMSECV and RMSEC were calculated for each model, in order to compare the predictive value and the fit of the different models, respectively. All steps involved in the activity modelling were conducted in Matlab.

## 4. Conclusions

The antiplasmodial and cytotoxic activities of ten cyclopeptide alkaloids were assessed, with the most promising result found for spinanine-B, with an IC_50_ value of 2.1 μM and without cytotoxic effects in concentrations up to 64.0 μM. Combining these results with those previously reported by our group for nine other cyclopeptide alkaloids, a qualitative structure-activity relationship study concerning the antiplasmodial activity was performed. The obtained results indicated that a 13-membered ring is preferred over a 14-membered ring, and that methoxylation in position 2 of the styrylamine moiety, which takes part in this ring, is beneficial, although hydroxylated compounds also displayed antiplasmodial activity. However, in order to confirm the importance of the methoxy group and the β-hydroxy proline moiety, and in order to determine the exact role of different aliphatic amino acids (leucine, isoleucine, valine) in either the macrocyclic ring or the side chain, further research is required.

For each of the tested cyclopeptide alkaloids, 75 molecular descriptors were calculated for their lowest energy conformer. In this way, a library with data on 19 cyclopeptide alkaloids was created, which was used to develop QSAR models based on PLS and MLR modelling. These QSAR models defined quantitative correlations between the molecular descriptors and the antiplasmodial activity, and indicated which molecular descriptors can explain the most variance regarding the antiplasmodial activity. These descriptors were, in order of decreasing importance: the Platt index, the MMFF94 energy, the Dreiding energy, the maximal and minimal projection area, the Wiener polarity, the percentage of C-atoms, and the aliphatic bond count. The obtained models can be applied for the prediction of the antiplasmodial activity of other cyclopeptide alkaloids. These predictions were often extrapolations relative to our small calibration set, but seem to confirm our qualitative SAR study (at least did not contradict the expectations).

## Figures and Tables

**Table 1:** Antiplasmodial activity against *P. falciparum* and cytotoxicity against MRC-5 cells (IC_50_ value in μM) for cyclopeptide alkaloids isolated from *Hymenocardia acida*, *Hovenia dulcis*, and different *Ziziphus* spp.

	Compound	Plant Source	*P. falciparum* K1 IC_50_ (μM)	MRC-5 IC_50_ (μM)
**1**	Nummularine-U	*Z. nummularia*	23.0 ± 8.5	>64.0
**2**	Mauritine-F		34.2 ± 9.14	>64.0
**3**	Spinanine-B	*Z. spina-christi*	2.1 ± 0.3	>64.0
**4**	Nummularine-E		>64.0	>64.0
**5**	Amphibine-D		8.9 ± 1.5	>64.0
**6**	Jubanine-F	*Z. jujuba*	12.8 ± 2.9	>64.0
**7**	Jubanine-G		4.7 ± 2.4	>64.0
**8**	Nummularine-B		3.6 ± 1.3	>64.0
**9**	Adouetine-X		7.5 ± 1.8	19.1 ± 11.9
**10**	Frangulanine	*H. dulcis*	14.9 ± 5.2	30.6 ± 6.5
**11**	Hymenocardine	*H. acida*	16.4 ± 6.8	51.1 ± 17.2
**12**	Hymenocardinol		17.5 ± 8.7	>64.0
**13**	Hymenocardine N-oxide		12.2 ± 6.6	>64.0
**14**	Hymenocardine-H		27.9 ± 16.5	>64.0
**15**	Nummularine-R	*Z. oxyphylla*	3.2 ± 2.6	30.6 ± 4.0
**16**	O-desmethylnummularine-R		7.1 ± 1.6	>64.0
**17**	Hemsine-A		13.6 ± 9.3	>64.0
**18**	Ramosine-A		>32.0	>64.0
**19**	Oxyphylline-F		7.4 ± 3.0	31.2 ± 1.4
Control 1	Chloroquine		0.15	nt
Control 2	Tamoxifen		nt	10.0

nt: not tested.
